# Draft Genome Sequence of *Clostridium mangenotii* TR, Isolated from the Fecal Material of a Timber Rattlesnake

**DOI:** 10.1128/genomeA.01107-13

**Published:** 2014-01-09

**Authors:** Richard W. McLaughlin, Philip A. Cochran, Scot E. Dowd, Kylie Andersen, Nichole Anderson, Rachel Brennan, Nicole Brook, Tracie Callaway, Kimberly Diamante, Annie Duberstine, Karla Fitch, Heidi Freiheit, Chantel Godlewski, Kelly Gorman, Mark Haubrich, Mercedes Hernandez, Amber Hirtreiter, Beth Ivanoski, Xochitl Jaminet, Travis Kirkpatrick, Jennifer Kratowicz, Casey Latus, Tiegen Leable, Nicole Lingafelt, DeAnna Lowe, Holly Lowrance, Latiffa Malsack, Julie Mazurkiewicz, Persida Merlos, Jamie Messley, Dawn Montemurro, Samora Nakitare, Christine Nelson, Amber Nye, Valerie Pazera, Gina Pierangeli, Ashley Rellora, Angelica Reyes, Jennifer Roberts, Shadara Robins, Jeshannah Robinson, Alissa Schultz, Sara Seifert, Elona Sigler, Julie Spangler, Ebony Swift, Rebecca TenCate, Jessica Thurber, Kristin Vallee, Jennifer Wamboldt, Shannon Whitten, De’andrea Woods, Amanda Wright, Darin Yankunas

**Affiliations:** General Studies, Gateway Technical College, Kenosha, Wisconsin, USAa; Biology Department, Saint Mary’s University of Minnesota, Winona, Minnesota, USAb; MR DNA (Molecular Research LP), Shallowater, Texas, USAc

## Abstract

Here, we report the draft genome sequence of *Clostridium mangenotii* strain TR, which was isolated from the fecal material of a timber rattlesnake. This bacterium is nonpathogenic but contains 68 genes involved in virulence, disease, and defense.

## GENOME ANNOUNCEMENT

*Clostridium mangenotii* is a Gram-positive rod that is capable of forming endospores. It is nonmotile and is a strict anaerobe, with an optimal growth temperature of 37°C. This bacterium is found in soil and has been isolated from African soil in particular (1). In this study, *C. mangenotii* strain TR was isolated from the fecal material of a timber rattlesnake using the ethanol shock method (2). The cells were plated on blood agar and incubated for 48 h under anaerobic conditions.

A colony of *C. mangenotii* was sent to Molecular Research LP (MR DNA [http://www.mrdnalab.com]). DNA was isolated using the Qiagen mini DNA isolation kit (Qiagen, Inc.). Afterward, using an Ion Express library kit and the Ion Torrent PGM sequencer (Life Technologies), a draft genome was sequenced according to the manufacturer’s guidelines. Using NGen (DNAStar), the genome was assembled, and annotation of the genome was performed using RAST (http://rast.nmpdr.org) (3).

In total, 82 contigs with protein-encoding genes (PEGs) were constructed, totaling 3,052,440 bp. Annotation in RAST revealed 146 RNAs, 3,093 coding sequences, and 323 subsystems. There were 68 genes involved in virulence, disease, and defense. Some of these genes are involved in vancomycin, fluoroquinolone, zinc, and arsenic resistance.

### Nucleotide sequence accession numbers.

This whole-genome shotgun project has been deposited at DDBJ/EMBL/GenBank under the accession no. AXUS00000000. The version described in this paper is version AXUS01000000.
